# Effect of Acupuncture on Delayed Emesis for the Patients Who Received High-Emetogenic Chemotherapy with Standard Antiemetic Prophylaxis (KHMC-HO-01): An Open-Label, Randomized Study

**DOI:** 10.1155/2022/9688727

**Published:** 2022-04-05

**Authors:** Chi Hoon Maeng, Seunghoon Lee, Jae Joon Han, Hong Jun Kim, Dongwoo Nam, Junhee Lee, Sun Kyung Baek

**Affiliations:** ^1^Division of Medical Oncology‐Hematology, Department of Internal Medicine, Kyung Hee University Hospital, Kyung Hee University College of Medicine, Seoul, Republic of Korea; ^2^Department of Acupuncture & Moxibustion, Kyung Hee University College of Korean Medicine, Seoul, Republic of Korea; ^3^Department of Sasang Constitutional Medicine, Kyung Hee University College of Korean Medicine, Seoul, Republic of Korea

## Abstract

**Background:**

Chemotherapy-induced nausea and vomiting (CINV) is one of the most important issues associated with chemotherapy. The additional or synergistic effect of acupuncture on CINV remains controversial.

**Methods:**

Patients were randomized into either the group that received standard antiemetics with acupuncture (Arm A) or standard antiemetics only (Arm C). Acupuncture with manual stimulation was applied at eight predefined points and was started before the first cycle of chemotherapy on the first day and two additional sessions were administered on the second day of chemotherapy. Acute and delayed CINV was assessed using the Rhodes Index of Nausea, Vomiting, and Retching (RINVR) and the MASCC Antiemesis Tool (MAT). The primary outcome was the delayed nausea score assessed using the RINVR.

**Results:**

Overall, 42 patients were included. In the delay phase, the severity of delayed nausea was slightly lower without significance in Arm A than in Arm C (5.35 vs. 5.98, *p* = 0.3011). Similarly, patients in Arm A reported less severe vomiting than those in Arm C (0.75 vs. 1.25, *p* = 0.3064). Delayed nausea and vomiting assessed by the MAT showed significant relief with acupuncture compared to standard antiemesis alone. In terms of acute emesis, there was no significant difference between the two arms according to either scoring method.

**Conclusions:**

Delayed nausea after HEC tended to decrease with acupuncture using the RINVR score, though it was also not significant. With the MAT assessment, delayed emesis (nausea and vomiting) was significantly improved with acupuncture, suggesting a promising effect of acupuncture. This trial is registered with KCT0006477.

## 1. Background

Chemotherapy-induced nausea and vomiting (CINV) is one of the most frequent and anxiety-producing side effects in patients receiving chemotherapy [1]. Poorly controlled CINV may adversely affect patients' quality of life and ability to perform basic activities of daily living. Furthermore, severe and uncontrolled emesis can lead to a delay in the administration of chemotherapy, a reduction in the dose, or even a patient's refusal of potentially life-saving treatment such as adjuvant chemotherapy [2]. Nausea, especially delayed CINV, is known as a particularly uncontrollable and distressing symptom that is significant despite the use of modern antiemetics, including 5-hydroxytryptamine (5-HT3) receptor and antagonists and neurokinin-1 (NK-1) receptor antagonists, and healthcare providers tend to underestimate its effects after both moderately and highly emetogenic chemotherapy (HEC) [3, 4].

Acupuncture-point stimulation has long been studied as a complementary and alternative treatment for CINV and encompasses manual acupuncture using needles, acupressure, electroacupuncture, and noninvasive electrostimulation. However, previous studies have frequently had small study populations, have been quite heterogeneous in terms of trial designs, and had inconsistent results [5]. Thus, the role of acupuncture in CINV remains controversial. A number of studies have not distinguished between the effects of acupuncture on acute versus delayed CINV or have only investigated the effects on acute CINV [5]. While a randomized clinical trial of CINV in breast cancer patients showed a reduction in delayed CINV, that study was not conducted using conventional manual acupuncture but rather applied acupressure [6]. A Cochrane review revealed that acupressure had a modest effect on the severity of acute nausea but not on delayed CINV [7, 8]. The studies included in the meta-analysis using manual acupuncture only tested the effect on acute CINV. Another more recent systematic review found that acupuncture may be useful in reducing CINV. However, it also reported that several studies had an unclear risk of bias, highlighting the need for additional studies [9]. It should be noted that most of these previous studies were conducted before the introduction of modern antiemetics, including NK-1 receptor antagonists. Considering the lack of solid evidence regarding acupuncture, especially for delayed nausea, in this study, we aimed to investigate whether conventional acupuncture would be effective for delayed CINV in patients receiving HEC along with standard antiemetic prophylaxis.

## 2. Methods

### 2.1. Patients

Patients aged ≥19 years who were chemotherapy-naïve and were set to receive HEC as adjuvant or first-line palliative treatment were eligible for inclusion. HEC was defined as cisplatin-containing more than 50 mg/m^2^ of the body surface area or a combination chemotherapy with doxorubicin and cyclophosphamide [4]. Concurrent chemoradiotherapy was not regarded as HEC. Patients with hematological malignancies were excluded, although any type of solid cancer was eligible. Other major criteria for inclusion were an Eastern Cooperative Oncology Group (ECOG) performance status between 0 and 2 and adequate organ function. Patients who had clinical conditions that could cause nausea and vomiting other than chemotherapy, such as bowel obstruction, active gastrointestinal ulcer, and brain metastasis, were excluded. In addition, patients who received any type of acupuncture or who were taking daily medication that had antiemetic effects, such as steroids or benzodiazepines, were also excluded. Finally, regardless of the reason, any subject who experienced nausea or vomiting in the week prior to study enrollment were also excluded to reduce bias.

### 2.2. Study Design

This study was an investigator-initiated, open-label, randomized, single-center, phase 2 clinical trial. Patients were randomly assigned at a 1 : 1 ratio to either the acupuncture arm (Arm A) or the control arm (Arm C) using permuted block randomization generated by an independent statistician from the investigators. The protocol was approved by the institutional review boards of both Kyung Hee University Hospital and Korean Medicine Hospital (IRB approval numbers KHUH 2014-03-204 and KOMCIRB 2014-71, respectively). The trial was carried out in accordance with the Declaration of Helsinki and Good Clinical Practice guidelines for Korea. Written informed consent was obtained from all the patients before study entry. This clinical trial was registered in the Clinical Research Information Service (KCT0006477), Korean Clinical Trial Registry.

### 2.3. Procedure

All patients received standard prophylactic antiemetics prior to the initiation of chemotherapy. Before chemotherapy was first initiated, patients allocated to Arm C (control arm) received oral aprepitant (125 mg), oral metoclopramide (three 10-mg doses), intravenous ramosetron (0.3 mg), and intravenous dexamethasone (12 mg). On days 2 and 3, oral aprepitant (80 mg), oral metoclopramide (three 10-mg doses), and intravenous dexamethasone (8 mg) were administered [10–12]. On day 4, oral metoclopramide (three 10-mg doses) and intravenous dexamethasone (8 mg) were administered. Finally, on day 5, three 10 mg doses of oral metoclopramide were administered. From the sixth day, rescue medications (metoclopramide or lorazepam) were available and administered based on the severity of the patient's symptoms. Patients assigned to Arm A (acupuncture) received manual acupuncture in addition to all the above antiemetic prophylaxis treatments. A total of three acupuncture sessions were performed using 0.25 × 40 mm disposable sterile acupuncture needles (Dongbang Acupuncture Inc., Chungnam, South Korea), which were retained for 20 min. The patients received acupuncture treatment 1 h before starting chemotherapy on the first day and then twice (morning and afternoon) the next day. Four acupuncture points, such as PC6, LI4, ST36, and LR3, were chosen bilaterally based on the previous literature [13] and the tonification method of spleen meridian (HT8, SP2, LR1, SP1) or the sedation method of stomach meridian (GB41, SI5, LI1, ST45) in Sa-Am acupuncture at the other side according to the determination of Korean medical doctors based on the traditional Korean medicine theory (a total of 8 acupuncture points) [14].

### 2.4. Assessment

To assess the CINV, we used two kinds of validated questionnaires, the Rhodes Index of Nausea, Vomiting, and Retching (RINVR) and the Multinational Association of Supportive Care in Cancer (MASCC) antiemetic tool (MAT). The RINVR is an evaluation tool developed by Rhodes to objectively measure the degree of nausea and vomiting in patients receiving chemotherapy and is divided into the survey for acute and delayed emesis, respectively [15]. The RINVR uses for questions 1 to 8 with a 5-point scale representing the severity of symptoms. For the raw score, less points are considered to have a better outcome. This tool has been validated in Korean populations [16]. The primary outcome was the intensity of delayed nausea, measured using the RINVR from 24 h after the initial administration of chemotherapy to 120 hours postadministration. Although the RINVR score was the primary outcome in this study, we also investigated similar parameters simultaneously using the MAT. The MAT is a simple questionnaire examining the degree of nausea and vomiting [17]. For nausea, the respondent was asked to indicate the presence or absence of nausea and the severity (if present) on a scale of 0–10. For vomiting, the presence or absence of vomiting and the number of vomiting during a given observation period are asked. Like RINVR, MAT is also divided into the survey for acute and delayed emesis according to the time of response. In order to reduce the participant's recall bias, the patient was asked to fill out each questionnaire every day as a form of a diary. The patient diary consisting of combined questionnaires is provided from the start of chemotherapy. If a patient is discharged from the hospital after completing chemotherapy, for the evaluation of delayed vomiting, check the condition by phone call daily during the observation period.

### 2.5. Statistical Analysis

The sample size was calculated based on the primary outcome. The patients were recruited and assigned to the treatment arms at a 1 : 1 ratio, the type 1 error was set at 0.05, and the type 2 error was set as 0.1. The sample size was determined by reference to a controlled before-and-after study that evaluated acute and delayed RINVR scores after PC6 acupressure among patients with gynecological cancer who were receiving chemotherapy [18]. In this study, a total of 3 measurements were taken on days 2, 3, and 4 after chemotherapy administration. The difference in the effect increased over time between the experimental and control groups. To calculate the sample size most conservatively despite the primary outcome being delayed symptoms, the standard deviation (*σ*) was set at 2.54 and the expected mean difference (d) was set at 2.7 based on the value measured on study day 2. Accordingly, 19 patients were required for each study arm [18]. Considering a drop rate of 10%, the total samples size was 44 people (22 in Arm A and 22 in Arm C). Fisher's exact test was used to confirm that baseline characteristics were evenly distributed between the two arms. For the analysis of the severity of nausea based on RINVR during the delayed phase, we identified the peak of the nausea score (the highest scores) that each individual patient answered during days 2–5 after chemotherapy based on the questionnaire. And then, the average of the highest scores for each subject was then compared between the two arms using analysis of covariance (ANCOVA). Considering the effect that the symptoms experienced on the first day of chemotherapy (acute phase) had on the severity of nausea during the subsequent period, the scores were adjusted according to the score on day 2. Other parameters of RINVR (vomiting, retching, and total score) were also analyzed using the same method. Repeated measures analysis of variance (RM ANOVA) was used to analyze the distribution of scores for each symptom. The severity of nausea or the frequency of vomiting based on the MAT was also analyzed using the average of the highest scores during the acute and delayed phases. The average scores were compared between the arms using the independent samples *t*-test or Wilcoxon's rank-sum test. Daily changes in the MAT scores were analyzed and compared between the arms using RM ANOVA. All statistical analyses were performed using PASW Statistics for Windows (version 18, SPSS Inc., Chicago, IL, USA).

## 3. Results

**Figure 1:**
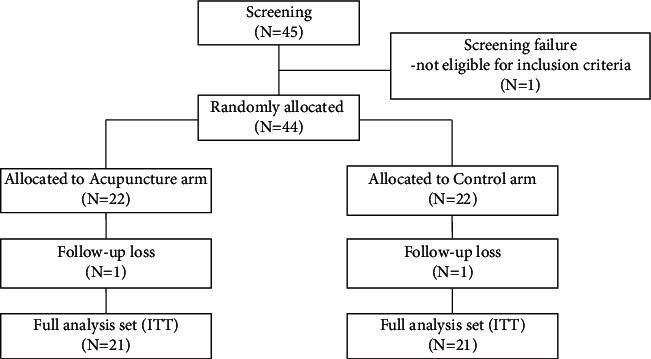
CONSORT diagram.

This study was conducted from March 2015 to March 2019 at the Department of Medical Oncology and Hematology at Kyung Hee University Hospital and the Department of Acupuncture and Moxibustion at Kyung Hee University Korean Medicine Hospital. A total of 45 patients agreed to participate in the study and provided informed consent. Among these, two patients dropped out of follow-up and one patient was excluded from the efficacy analysis due to a major violation of the inclusion criteria. Therefore, a total of 42 patients (21 in each arm) were included in the analysis.  Table 1 summarizes the demographic characteristics of the study population (Figure 1). Each variable was evenly distributed between the two arms. Most of the participants had breast cancer and were receiving a combination of anthracycline and cyclophosphamide (AC) as neoadjuvant or adjuvant chemotherapy.

### 3.1. Outcomes Based on RINVR Score

**Figure 2:**
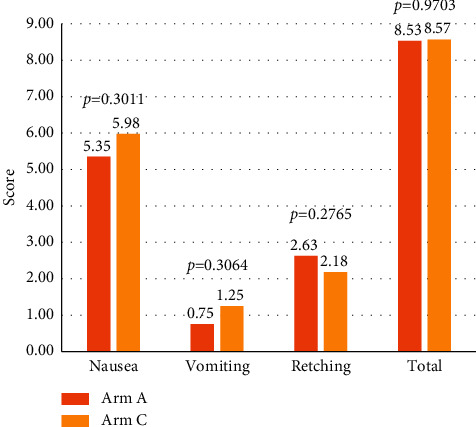
Outcomes based on RINVR score in the delayed phase.

**Figure 3:**
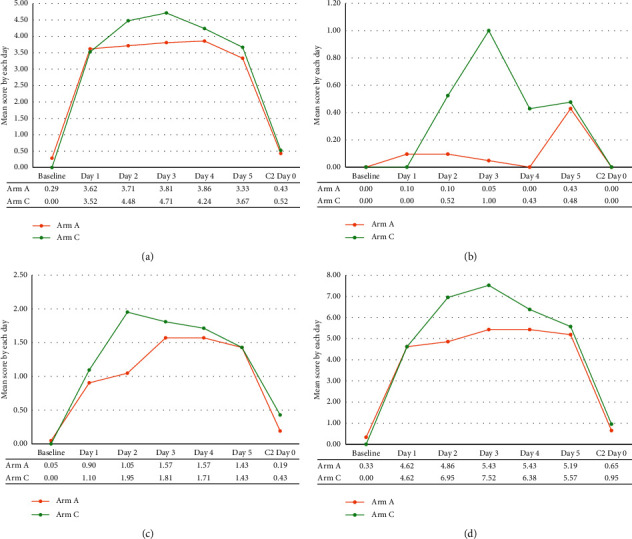
The pattern of changes in outcome according to each day after chemotherapy (RINVR). (a) Nausea, (b) vomiting, (c) retching, and (d) total.

In the delayed phase, the severity of delayed nausea was slightly lower in Arm A than that in Arm C, although the difference was not statistically significant (Arm A: 5.35 vs. Arm C: 5.98, *p* = 0.3011, Figure 2). Similarly, patients in Arm A reported less severe vomiting in the delayed phase than those in Arm C, though the difference was not statistically significant (Arm A: 0.75 vs. Arm C: 1.25, *p* = 0.3064, Figure 2). The retching score was higher in Arm A than that in Arm C (Arm A: 2.63 vs. Arm C: 2.18, *p* = 0.2765, Figure 2). Similar patterns were seen when comparing total RINVR scores, with lower total scores (i.e., less symptomatic) in Arm A, though the difference was negligible and not statistically significant (Arm A: 8.53 vs. 8.57, *p* = 0.9703, Figure 2). The RINVR scores in the acute phase were not different between the two arms in terms of nausea, vomiting, retching, or total scores. Indeed, the differences between the two arms were almost negligible (most were ≤0.1 points) and were not statistically significant (Supplementary Table S1 and Figure S1). When comparing the mean scores according to the number of days postchemotherapy administration, the differences in nausea, vomiting, retching, and total scores trended towards being more prominent on days 2–4 (Figure 3 and Table S2). However, there was no difference on day 1 (acute phase). Of note, the mean vomiting score on day 4 was significantly lower in Arm A than that in Arm C (Arm A: 0.00 vs. Arm C: 0.43, *p* = 0.0250) (Figure 3(b)).

### 3.2. Outcomes Based on MAT

**Figure 4:**
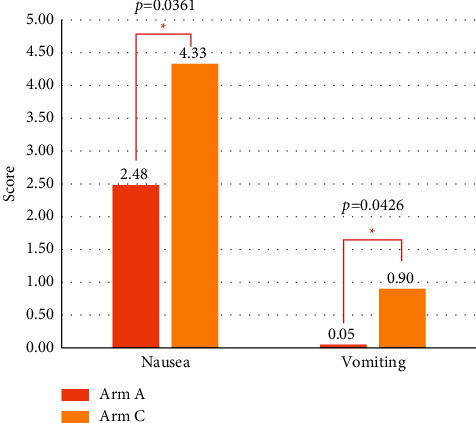
Outcomes based on MAT in the delayed phase.

**Figure 5:**
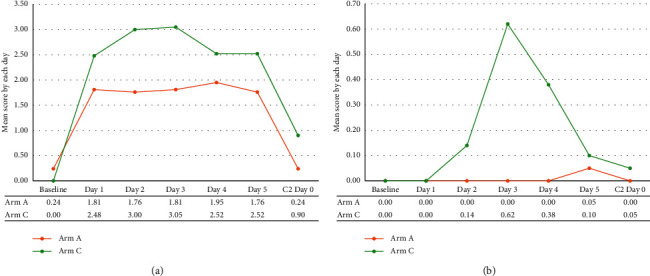
The pattern of changes in outcome according to each day after chemotherapy (MAT). (a) Nausea and (b) vomiting.

In the delayed phase, patients in Arm A showed significantly lower nausea scores than those in Arm C (Arm A: 2.48 vs. 4.33, *p* = 0.0361, Figure 4 and Table S3). The vomiting score was also lower in Arm A than that in Arm C, with statistical significance (Arm A: 0.05 vs. Arm C: 0.90, *p* = 0.0426, Figure 4 and Table S3). Similar to the RINVR results, there was no difference in the vomiting score in the acute phase (i.e., the number of vomiting episodes on day 1) between the two arms. In fact, the reported number of vomiting episodes in each arm was 0, indicating that neither group had any episodes of vomiting. When comparing the mean scores according to the number of days postchemotherapy administration, the differences in nausea and vomiting tended to be more prominent on days 2–4 after chemotherapy, although the difference was not statistically significant (Figure 5 and Table 4S).

### 3.3. Safety

There was no acupuncture-related adverse event. None of the participants showed bleeding, local irritation, or infection. Although most participants complained of very transient and mild cutaneous pain at the start of needling, we did not regard this as an adverse event.

## 4. Discussion

The mechanism through which acupuncture affects CINV has not been fully elucidated [5]. Some potential explanations are that acupuncture mediates the regulation of the gastrointestinal site of action of 5-HT and endocannabinoid release and reduces the levels of plasma monoamine neurotransmitters 5-HT [19]. A recent study reported that electroacupuncture decreased the serum levels of 5-HT and dopamine in patients who received highly or moderately emetogenic chemotherapy [20]. Similarly, the electroacupuncture performed in that study showed effects in the delayed phase but not in the acute phase. However, considering that 5-HT is a neurotransmitter mainly involved in acute rather than delayed emesis, further studies are needed to define the relationship more clearly between serum 5-HT concentrations and the acupuncture-mediated effect on delayed emesis that could directly explain the mechanism of action. In our study, acupuncture did not result in additional improvements in the severity of nausea in the delayed phase. However, although there was no statistical significance, a meaningful and interesting trend indicating that acupuncture was effective in alleviating delayed nausea and vomiting was consistently observed using a number of parameters. Briefly, the degree of nausea and the number of vomiting episodes were less severe in Arm A than those in Arm C. Of note, the outcomes reported by MAT showed statistically significant differences in the effect of acupuncture. These effects were not observed during the acute phase. Recently, a randomized controlled study reported that acupuncture had a modest effect on reducing the severity of nausea and vomiting based on the Common Terminology Criteria for Adverse Events version 4.0 (CTCAE v4.0) in the delayed phase but not in the acute phase without statistical significance [21]. Similar to our findings, electronic acupuncture had a significant effect on nausea from the third day and on vomiting from the fourth day after the chemotherapy session compared to the sham acupuncture group, even though the complete response rate, which was the primary outcome, was not statistically different. In our study, daily reported scores showed that the severity of nausea and vomiting was reduced from the second day of the chemotherapy session according to both the RINVR and MAT scores. Overall, the symptom score reduction in the acupuncture group was most pronounced from day 2–4 in our study. Likewise, acupressure has been shown to reduce delayed nausea and vomiting in patients with breast cancer [6].

Despite this similar trend, the lack of statistical significance in our study, unlike in other studies, can be attributed to differences in study design, including the type of acupuncture (manual vs. electronic vs. acupressure), details regarding the procedure (body site, frequency, and duration of acupuncture), and outcome measurements (RINVR/MAT vs. CTCAE). In a previous systematic review, conflicting results in two large studies [22, 23] were explained by differences in the acupuncture dose [7]. In the trial showing positive results [22], a total of five sessions of electroacupuncture were performed, whereas in the trial showing negative results [23], only two sessions of manual acupuncture were conducted. Although electroacupuncture reduced the incidence of acute vomiting, manual acupuncture did not. In our study, in which electrical stimulation was not used, manual acupuncture was administered a total of 3 times for 20 min per treatment because patients were hospitalized for chemotherapy for 2 or 3 days. It was considered inappropriate for participants to stay for a longer period for the study only. However, this procedure was thought to be suboptimal and, consequently, was not sufficient to achieve a significant effect.

Another important reason for the lack of statistical significance in our study may have been the use of modern antiemetics. With the introduction of neurokinin-1 receptor antagonists (NK-1RA), such as aprepitant, acute and delayed CINV has been significantly reduced. Most previous studies evaluating the effect of acupuncture on CINV have not used NK-1RA in the control arm. Recent guidelines strongly recommend the combination of antiemetic prophylaxis, including NK-1RAs, 5-HT3 receptor antagonists, and glucocorticoids with or without olanzapine, especially in patients who receive HEC [4, 24]. To the best of our knowledge, no clinical study has investigated the effect of acupuncture along with NK-1RA, a 5-HT3 receptor antagonist, and a standard dose of dexamethasone compared to a control group that was also administered these antiemetics. Furthermore, all patients were administered metoclopramide for 5 days, which is approved as prophylaxis for delayed emesis in Korea. Although metoclopramide is not recommended as prophylaxis in the recent guidelines, it aids with managing delayed or breakthrough emesis [4, 25, 26]. Since our study focused on the delayed phase, we decided to use a combination of all available drugs that can prevent and/or relieve possible symptoms. Of note, no patients reported experiencing vomiting in either arm during the acute phase. Thus, it is possible to assume that the substantially improved antiemetic effect of this four-drug regimen (aprepitant, ramosetron, dexamethasone, and metoclopramide) for all enrolled patients regardless of the allocated arm might have diluted the additional beneficial effects of acupuncture.

After our study was initiated, palonosetron, a newer class of 5-HT3 receptor antagonist, has been widely established in clinical practice. Palonosetron is characterized by a long half-life and stronger binding affinity than other 5-HT3 receptor antagonists, including ondansetron, dolasetron, and granisetron [27]. Numerous studies have demonstrated that palonosetron showed superior efficacy than other 5-HT3 receptor antagonists [28–30]. In addition, a meta-analysis reported that there was no significant difference in side effects such as constipation, headache, diarrhea, and dizziness. It is also known to be safe in terms of acute arrhythmogenic potential. In our study, ramosetron was used instead of palonosetron [31]. However, in a recent prospective randomized clinical trial that compared ramosetron and palonosetron in patients receiving HEC in Korea, it was found that ramosetron was not inferior to palonosetron in both efficacy and safety and the quality of life [32]. Therefore, our study results will be meaningfully applied to the current daily practice. Although significant improvements in the effects of recent antiemetics have been evident, poorly controlled CINV is still a major unmet need. Acupuncture is relatively simple, inexpensive, and safe. For patients with insufficient emesis control despite standard antiemetic prophylaxis, it would be reasonable to actively consider adding acupuncture as an adjunct therapy.

Our study has several limitations. First, we did not use sham acupuncture since this was an open-label study. Sham acupuncture uses a placebo needle designed not to penetrate the skin or inserted at the wrong acupuncture points. Unlike a modern concept of randomized double-blinded studies, however, the role of sham puncture in acupuncture trials remains controversial. This is because, even with sham acupuncture, some acupressure may occur, and studies have reported that it can lead to efficacy [33, 34]. Second, among the participants, AC-based treated patients dominated, while less than 15% received cisplatin-containing chemotherapy. The proportion of patients treated with palliative chemotherapy was negligible in our study population. These are considered to limit the generalizability of the study's results. Nonetheless, the ratio of the participant distribution between arms A and C was not statistically different. Third, the delivered acupuncture dose seems to be insufficien and, thus, may be suboptimal to induce a significant effect. Since symptom severity differed the most between groups after day 3, it would be meaningful to examine the effect of acupuncture sustained for more than three days after chemotherapy in future studies.

## 5. Conclusions

In conclusion, performing manual acupuncture as an adjunct to standard antiemetic prophylaxis in patients receiving HEC did not significantly improve the severity of emesis compared to an antiemetic prophylaxis regimen alone. Nonetheless, according to the observed positive effect on delayed nausea, acupuncture could be an option that clinicians may consider according to their clinical judgment. Further well-designed studies of optimal electroacupuncture regimens are required to confirm the effectiveness of acupuncture for delayed CINV in patients receiving HEC along with standard antiemetic prophylaxis.

## Figures and Tables

**Table 1:** Baseline characteristics.

		Arm A		Arm C		*p* value
		N	%	N	%	
Age	<65	11	55.0	11	55.0	1.000
	>65	10	45.0	10	45.0	
	Median (year)	64		64		
Sex	M^a)^	2	9.5	2	9.5	1.000
	F^b)^	19	90.5	19	90.5	
Alcohol	No	21	100	20	95.2	1.000
	Yes	0	0	1	4.8	
ECOG PS^c)^	Grade 0	5	23.8	6	28.6	1.000
	Grade 1	16	76.2	15	74.4	
Comorbidity	No	12	57.1	15	71.4	1.000
	Yes	9	42.9	6	28.6	
Chemotherapy	AC^d)^-based	18	85.7	19	90.5	1.000
	Cisplatin-based	3	14.3	2	9.5	
Aim of treatment	Neoadjuvant	8	38.1	7	33.3	1.000
	Adjuvant	13	61.9	13	61.9	
	Palliative	0	0	1	4.8	

^*∗*^(a) M, male; (b) F, female; (c) ECOG, Eastern Cooperative Oncology Group performance status; (d) AC, adriamycin (doxorubicin) and cyclophosphamide.

## Data Availability

Data are contained within the article and are available on request.

## Abbreviations

CINV: Chemotherapy-induced nausea and vomiting
5-HT3: 5-Hydroxytryptamine
NK-1: Neurokinin-1
HEC: Highly emetogenic chemotherapy
ECOG: Eastern Cooperative Oncology Group
RINVR: Rhodes Index of Nausea, Vomiting, and Retching
MASCC: Multinational Association of Supportive Care in *Cancer*
MAT: MASCC antiemetic tool
ANCOVA: Analysis of covariance
RM ANCOVA: Repeated measures analysis of variance
AC: Anthracycline and cyclophosphamide
CTCAE v4.0: Common terminology criteria for adverse events version 4.0
NK1RA: Neurokinin-1 receptor antagonists.

## Supplementary Materials

Supplementary Materials File 1: Supplementary Tables. 1. Table S1 Score based on RINVR in the acute phase. 2. Table S2. The pattern of changes in outcome according to each day after chemotherapy (RINVR). 3. Table S3. Score based on MAT in the delayed phase. 4. Table S4. The pattern of changes in outcome according to each day after chemotherapy (MAT). File 2: Supplementary Figure. 1. Figure S1. Outcomes based on RINVR score in the acute phase. File 3: CONSORT checklist CONSORT 2010 guideline. This is a checklist proving that this study and the manuscripts complied with the CONSORT 2010 guidelines.
