# Supplementary material for: Effect of Acupuncture on Delayed Emesis for the Patients Who Received High-Emetogenic Chemotherapy with Standard Antiemetic Prophylaxis (KHMC-HO-01): An Open-Label, Randomized Study
Source: Evid Based Complement Alternat Med. 2022 Apr 5;2022:9688727. doi: 10.1155/2022/9688727 (PMC9005265; doi:10.1155/2022/9688727)
Supplement: Supplementary Materials — File 1: Supplementary Tables. 1. Table S1 Score based on RINVR in the acute phase. 2. Table S2. The pattern of changes in outcome according to each day after chemotherapy (RINVR). 3. Table S3. Score based on MAT in the delayed phase. 4. Table S4. The pattern of changes in outcome according to each day after chemotherapy (MAT). File 2: Supplementary Figure. 1. Figure S1. Outcomes based on RINVR score in the acute phase. File 3: CONSORT checklist CONSORT 2010 guideline. This is a checklist proving that this study and the manuscripts complied with the CONSORT 2010 guidelines. [file 9688727.f1.zip › 9688727.f1/[Proof-reading] Supplementary file lists and captions (20220323).docx]

# Supplementary file lists and captions

**File 1: Supplementary Tables**

1. Table S1

Score based on RINVR in acute phase

2. Table S2

The pattern of changes in outcome according to each day after chemotherapy (RINVR)

3. Table S3

Score based on MAT in delayed phase

4. Table S4

The pattern of changes in outcome according to each day after chemotherapy (MAT)

**File 2: Supplementary Figure**

1. Figure S1. Outcomes based on RINVR score in acute phase

**File 3: CONSORT checklist CONSORT 2010 guideline**

This is a checklist proving that this study and the manuscripts complied with the CONSORT 2010 guidelines.
