# Supplementary material for: Effect of Acupuncture on Delayed Emesis for the Patients Who Received High-Emetogenic Chemotherapy with Standard Antiemetic Prophylaxis (KHMC-HO-01): An Open-Label, Randomized Study
Source: Evid Based Complement Alternat Med. 2022 Apr 5;2022:9688727. doi: 10.1155/2022/9688727 (PMC9005265; doi:10.1155/2022/9688727)
Supplement: Supplementary Materials — File 1: Supplementary Tables. 1. Table S1 Score based on RINVR in the acute phase. 2. Table S2. The pattern of changes in outcome according to each day after chemotherapy (RINVR). 3. Table S3. Score based on MAT in the delayed phase. 4. Table S4. The pattern of changes in outcome according to each day after chemotherapy (MAT). File 2: Supplementary Figure. 1. Figure S1. Outcomes based on RINVR score in the acute phase. File 3: CONSORT checklist CONSORT 2010 guideline. This is a checklist proving that this study and the manuscripts complied with the CONSORT 2010 guidelines. [file 9688727.f1.zip › 9688727.f1/[Proof-reading] Supplementary Table 1-4.docx]

# S1 Table

**Score based on RINVR in acute phase**

|  | **Arm A** | **Arm C** | **p-value** |
| --- | --- | --- | --- |
| **Nausea** | 3.60 | 3.54 | 0.9613 |
| **Vomiting** | 0.10 | 0.00 | 0.3233 |
| **Retching** | 0.93 | 1.07 | 0.8066 |
| **Total** | 4.64 | 4.59 | 0.9754 |

Abbreviations: RINVR, the Rhodes Index of Nausea, Vomiting, and Retching

# S2 Table

**The pattern of changes in outcome according to each day after chemotherapy (RINVR)**

| Nausea |  |  |  |  |  |
| --- | --- | --- | --- | --- | --- |
| **Time point** | **Arm A** | | **Arm C** | | ***p* value** |
|  | **Mean** | **SD** | **Mean** | **SD** |  |
| Baseline | 0.29 | 0.90 | 0.00 | 0.00 | 0.1697 |
| Day 1 | 3.62 | 3.19 | 3.52 | 3.83 | 0.7965 |
| Day 2 | 3.71 | 2.55 | 4.48 | 3.60 | 0.5947 |
| Day 3 | 3.81 | 2.71 | 4.71 | 4.41 | 0.7230 |
| Day 4 | 3.86 | 2.87 | 4.24 | 3.88 | 0.9395 |
| Day 5 | 3.33 | 2.61 | 3.67 | 3.60 | 0.9290 |
| C2 Day 0 | 0.43 | 1.08 | 0.52 | 1.36 | 0.9672 |
| **Vomiting** |  |  |  |  |  |
| **Time point** | **Arm A** | | **Arm C** | | ***p* value** |
|  | **Mean** | **SD** | **Mean** | **SD** |  |
| Baseline | 0.00 | 0.00 | 0.00 | 0.00 | 1.0000 |
| Day 1 | 0.10 | 0.44 | 0.00 | 0.00 | 0.3465 |
| Day 2 | 0.10 | 0.30 | 0.52 | 1.83 | 0.9413 |
| Day 3 | 0.05 | 0.22 | 1.00 | 2.35 | 0.1476 |
| Day 4 | 0.00 | 0.00 | 0.43 | 0.98 | ***0.0250**** |
| Day 5 | 0.43 | 1.21 | 0.48 | 0.98 | 0.5123 |
| C2 Day 0 | 0.00 | 0.00 | 0.00 | 0.00 | 1.0000 |
| **Retching** |  |  |  |  |  |
| **Time point** | **Arm A** | | **Arm C** | | ***p* value** |
|  | **Mean** | **SD** | **Mean** | **SD** |  |
| Baseline | 0.05 | 0.22 | 0.00 | 0.00 | 0.3465 |
| Day 1 | 0.90 | 1.70 | 1.10 | 2.02 | 0.9404 |
| Day 2 | 1.05 | 1.66 | 1.95 | 2.33 | 0.1020 |
| Day 3 | 1.57 | 1.80 | 1.81 | 2.62 | 0.6259 |
| Day 4 | 1.57 | 1.69 | 1.71 | 2.31 | 0.9057 |
| Day 5 | 1.43 | 1.66 | 1.43 | 2.06 | 0.7117 |
| C2 Day 0 | 0.19 | 0.60 | 0.43 | 1.43 | 0.9413 |
| **Total (Nausea+Vomiting+Retching)** | | |  |  |  |
| **Time point** | **Arm A** | | **Arm C** | | ***p* value** |
|  | **Mean** | **SD** | **Mean** | **SD** |  |
| Baseline | 0.33 | 1.06 | 0.00 | 0.00 | 0.1698 |
| Day 1 | 4.62 | 4.55 | 4.62 | 5.29 | 0.8873 |
| Day 2 | 4.86 | 3.92 | 6.95 | 5.95 | 0.2619 |
| Day 3 | 5.43 | 4.15 | 7.52 | 7.41 | 0.5219 |
| Day 4 | 5.43 | 4.03 | 6.38 | 6.05 | 0.8503 |
| Day 5 | 5.19 | 4.91 | 5.57 | 5.39 | 1.0000 |
| C2 Day 0 | 0.65 | 1.42 | 0.95 | 2.60 | 0.7669 |

Italic *p*-value designated as * indicates statistical significance using Repeated Measures Analysis of variance.

Abbreviations: SD, standard deviation; C1, cycle 1; C2, cycle 2.

# S3 Table

**Score based on MAT in delayed phase**

|  | **Arm A** | **Arm C** | ***p*-value** |
| --- | --- | --- | --- |
| **Nausea** | 2.48 | 4.33 | *0.0361** |
| **Vomiting** | 0.05 | 0.90 | *0.0426** |

Italic *p*-value designated as * indicates statistical significance using Repeated Measures Analysis of variance.

Abbreviations: MAT; the Multinational Association of Supportive Care in Cancer (MASCC) antiemetic tool

# S4 Table

**The pattern of changes in outcome according to each day after chemotherapy (MAT)**

| **Nausea** |  |  |  |  |  |
| --- | --- | --- | --- | --- | --- |
| **Time point** | **Arm A** | | **Arm C** | | ***p* value** |
|  | **Mean** | **SD** | **Mean** | **SD** |  |
| Baseline | 0.24 | 1.09 | 0.00 | 0.00 | 0.3465 |
| Day 1 | 1.81 | 1.91 | 2.48 | 3.08 | 0.7853 |
| Day 2 | 1.76 | 1.48 | 3.00 | 2.55 | 0.1392 |
| Day 3 | 1.81 | 1.63 | 3.05 | 2.80 | 0.1997 |
| Day 4 | 1.95 | 1.88 | 2.52 | 2.80 | 0.7116 |
| Day 5 | 1.76 | 1.84 | 2.52 | 2.98 | 0.7981 |
| C2 Day 0 | 0.24 | 0.54 | 0.90 | 2.00 | 0.5089 |
| **Vomiting** |  |  |  |  |  |
| **Time point** | **Arm A** | | **Arm C** | | ***p* value** |
|  | **Mean** | **SD** | **Mean** | **SD** |  |
| Baseline | 0.00 | 0.00 | 0.00 | 0.00 | 1.0000 |
| Day 1 | 0.00 | 0.00 | 0.00 | 0.00 | 1.0000 |
| Day 2 | 0.00 | 0.00 | 0.14 | 0.65 | 0.3465 |
| Day 3 | 0.00 | 0.00 | 0.62 | 1.83 | 0.0882 |
| Day 4 | 0.00 | 0.00 | 0.38 | 1.16 | 0.0882 |
| Day 5 | 0.05 | 0.22 | 0.10 | 0.30 | 0.5760 |
| C2 Day 0 | 0.00 | 0.00 | 0.05 | 0.22 | 0.3465 |

Abbreviations: SD, standard deviation; C1, cycle 1; C2, cycle 2.
